# Effects of the COVID-19 Pandemic on Head and Neck Cancer Stage and Treatment Duration

**DOI:** 10.7759/cureus.26744

**Published:** 2022-07-11

**Authors:** Savvas Kourtidis, Julia Münst, Veit M Hofmann

**Affiliations:** 1 Department of Otorhinolaryngology - Head and Neck Surgery, Charité Universitätsmedizin Berlin, Berlin, DEU

**Keywords:** delay, tnm, staging, head and neck cancer, pandemic, covid-19

## Abstract

Objective

To assess the efficacy of oncologic healthcare during the COVID-19 pandemic on patients with head and neck squamous cell carcinoma (SCC) in a tertiary university hospital in Germany.

Methods

This retrospective, cross-sectional, observational study included 94 patients with newly diagnosed head and neck squamous cell carcinoma during a two-year period. Patients were assigned to two date-dependent groups; referrals before (group A) and during (group B) the COVID-19 pandemic. Time intervals from the symptom(s) onset to diagnosis, diagnosis to treatment, and treatment initiation to completion were recorded. Furthermore, TNM stages and the application of reconstructive surgery with free tissue transfer were determined. Patients’ outcomes and characteristics were compared between the two groups. Finally, a comprehensive literature review was carried out to identify similar epidemiological studies.

Results

The symptom-to-diagnosis interval was longer during the COVID-19 pandemic [median 9.5 (A) versus 15 (B) weeks, p = 0.054]. The intervals from diagnosis to treatment and treatment initiation to end of treatment were approximately the same in both groups [median 3 (A) versus 3.2 (B) weeks, p = 0.264; and 6.9 (A) versus 6.3 (B) weeks, p = 0.136]. The T-and N-stages were not higher during the pandemic [early T-stage (T1+T2) versus advanced T-stage (T3+T4), p = 0.668; and N-negative (N0) versus N-positive status (N1,2,3), p = 0.301]. Patients who presented with distant metastatic disease and those who underwent reconstructive surgery with free tissue transfer were observed more frequently in the lockdown phase [M1 versus M0, p= 0.022; and flap versus no flap, p=0.007].

Conclusion

This study suggests the consistent diagnostic and therapeutical performance of the tertiary oncologic healthcare in Berlin, Germany, despite the challenges that patient care units faced during the COVID-19 pandemic.

## Introduction

In March 2020, the World Health Organization (WHO) declared the global spread of the severe acute respiratory syndrome Coronavirus type 2 (SARS-CoV-2) as a pandemic [1]. Since then, healthcare institutions around the world have faced extraordinary challenges, and their consequences are still evident in every day's clinical practice [2]. The imposed radical governmental restrictions to combat the pandemic and the overstrained medical resources affected both physically and mentally, not only those infected by the novel virus but also miscellaneous patient groups [3]. Early reports highlighted a decline in cancer screening programs [4] and fewer cancer diagnoses [5] during the first months of the virus outbreak. Head and neck cancer (HNC) patients were not left unaffected by the circumstances. Therefore, multiple national associations and international consensus boards counteracted with altered cancer management protocols [6-8]. As the pandemic is still ongoing, experts are estimating the long-term compromise of cancer-specific mortality and survival rates [9].

The primary aim of this study is to evaluate the management efficacy of HNC patients during the Coronavirus disease 2019 (COVID-19) pandemic and address two main questions: did we notice any delays in referrals, diagnoses, and treatment times? Did patients present with higher tumor stages or more advanced locoregional/distal metastases?

The secondary aim is to provide the results from current epidemiological trials related to the same subjects in order to get insights into how patients with HNC were being cared for during the COVID-19 era.

## Materials and methods

Study design and ethics

**Figure 1:**
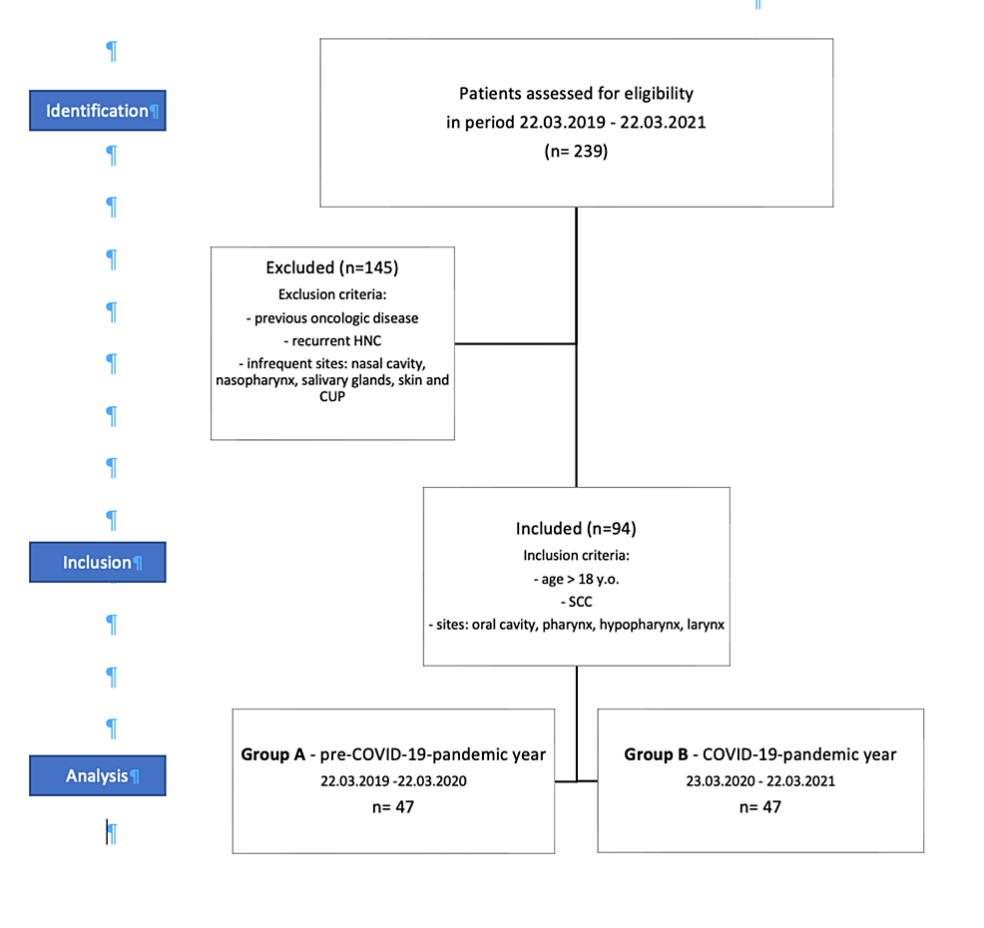
Patients selection flowchart.

This trial was conducted as a mono-centric, retrospective, cross-sectional observational study in accordance with the Strengthening the Reporting of Observational Studies in Epidemiology (STROBE) guidelines (Figure 1) that have been approved by our university's ethics committee. The study followed the principles of the International Conference on Harmonization: "Good Clinical Practice guidelines" as well as the revised version (2013) of the "Declaration of Helsinki." All patients’ clinical data have been treated anonymously and used only for research and scientific purposes.

Patients’ selection and stratification

The study’s cohort was selected from 239 consecutive patients who presented with head and neck malignancies in the Otorhinolaryngology-Head and Neck Surgery Department of Charité University Hospital, Berlin, from March 22, 2019 to March 22, 2021. This research period was divided into two equal subperiods of one year each and comprised of patients who were diagnosed before (group A) and after (group B) the federal state’s imposition of lockdown on March 22, 2021 (Figure 1). The inclusion criteria were age >18 years old, squamous cell carcinoma (SCC) cases of the oral cavity, pharynx, hypopharynx, or larynx, with no previously known oncologic disease of any kind, or recurrent head and neck cancer. Patients with malignancies other than SCC, such as lymphoma, skin cancer, salivary gland malignancies, adenocarcinoma, and the manifestation of tumors in infrequent sites such as the nasal cavity, paranasal sinuses, and cancer of unknown primary (CUP), were excluded. Thereupon, 78 and 67 patients were excluded from groups A and B, respectively, resulting coincidentally in two equal groups of 47 cases each.

Data acquisition

Patients’ clinical data were obtained using SAP Patient Management software (SAP, Walldorf, Germany). In particular, demographic characteristics (age, sex, etc.), site of cancer (oral cavity, pharynx, hypopharynx, and larynx), symptom-diagnosis interval (SDI - time between the first symptom related to cancer manifestation and diagnosis through biopsy), diagnosis-treatment interval (DTI - time between diagnosis through histological verification of SCC and initiation of therapy, either radiotherapy or surgery or combination), treatment-end-of-treatment interval (TEI - time between initiation of treatment and the definitive end of the therapeutical concept), clinical/pathological TNM-stage (according to the 8th edition of the Union for International Cancer Control TNM classification) and free tissue transfer reconstruction (for example, radial forearm flap) were registered.

A special focus was laid on recording meticulously the time and type of each diagnostic/therapeutical step. The therapeutical concepts consisted of surgical resection only, the surgical procedure followed by adjuvant radio(chemo)therapy [R(C)T] as well as primary R(C)T. Drop-outs after diagnostics or after receiving part of the therapeutical procedure were also recorded.

Statistical analyses

The statistical analyses were performed at the Institute of Biometry and Clinical Epidemiology of the Charité-Universitätsmedizin Berlin (iBikE) using IBM SPSS Statistics V.24 (IBM, Armonk, NY). The Shapiro-Wilk test was used to analyze the distribution of all variables, while Pearson's chi-square test was used for comparisons of categorical and the Mann-Whitney U tests of numerical data. The global level of significance was set at α=0.05.

Comprehensive literature review

A comprehensive literature review was conducted according to PRISMA guidelines. The aim was to identify epidemiological observational studies that quantified treatment delays and upstage head and neck cancer during the COVID-19 pandemic. The PubMed/MEDLINE and Cochrane Library databases were used. The search terms "COVID-19," "coronavirus," "pandemic," "head and neck cancer," "oncologic patients," "treatment delay," "tumor size," and "upstage" were identified in 31 articles published from January 2020 to January 2022. The search strategy extended to the references of the identified articles, and 12 were chosen for full-text review. Seven articles reported quantitative measurements and, thus, met the inclusion criteria.

## Results

**Figure 2:**
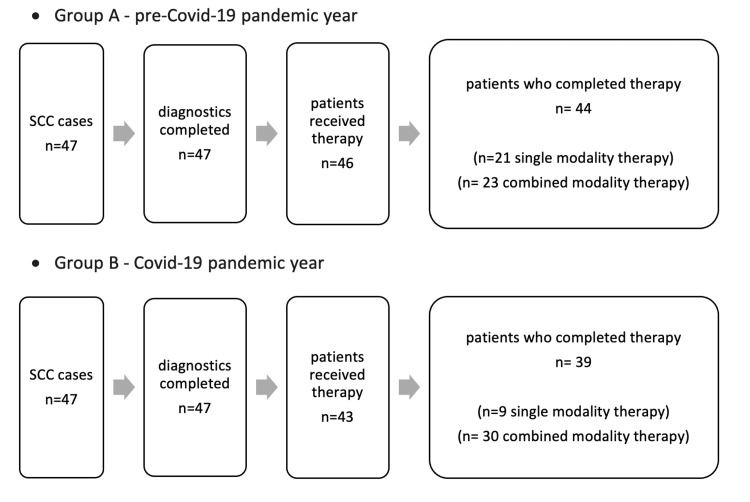
Progressive steps from diagnosis to therapy as well as definitive completion of therapeutical concept.

Figure 2 shows patients proceeding to each diagnostic and therapeutic step until definitive completion of the therapeutic concept in each group, respectively. In group A, one patient denied therapeutical intervention and two patients decided against adjuvant therapy for intrinsic reasons. In group B, four patients denied therapeutical intervention, three patients decided against completing the therapeutical concept after initial surgery, and one patient could not complete the adjuvant therapy because of COVID-19 infection.

Table 1 shows the demographics, tumor site, time intervals for each diagnostic/therapeutic step, TNM stages, therapeutic modalities, and free-flap reconstruction cases. Despite longer SDI during the COVID-pandemic (median 9.5 versus 15 weeks), there is no statistically significant difference between the two groups (p=0.054). DTI and TEI were approximately the same in both groups and did not show a statistically significant difference either. Furthermore, patients did not present with higher T-stages in the pandemic nor did they have more locoregional affection of lymph nodes (N+ status). Distant metastatic disease was more frequent during the COVID-19 pandemic (5 versus 0 cases, p=0.022) and surgical procedures with free-flap reconstruction (20 versus 12 cases, p=0.007) significantly increased.

**Table 1:** Summary of clinical data and statistical tests. Statistically significant p < 0.05 values are shown in bold NND: not normally distributed values, R(C)T: radio(chemo)therapy

Variables	Group A: pre-Covid-19	Group B: Covid-19	P-value (test)
Patients	47	47	
Sex	33♂/14♀	36♂/11♀	
Age	67.4 ± 10.6	69 ± 10.7	
Tumor site			
Oral cavity	5 (11%)	3 (6%)	
Oropharynx	19 (40%)	28 (60%)	
Larynx	19 (40%)	9 (19%)	
Hypopharynx	4 (9%)	7 (15%)	
SDI (weeks)			0.054 (Mann-Whitney-U-Test)
Median (Q1-Q3)	9.5 (4.5–20.5)	15 (6–27.1)	
Mean ± SD	15.6 ± 16.8	20.5 ± 17.2	
Range	0.5–75.5	1.8–77.4	
DTI (weeks)			0.264 (Mann-Whitney-U-Test)
Median (Q1-Q3)	3 (2.2–4)	3.2 (2.2–4.7)	
Mean ± SD	3.5 ± 2.7	3.8 ± 1.9	
Range	0.7–19	09–10.6	
TEI (weeks)			0.136 (Mann-Whitney-U-Test)
Median (Q1-Q3)	6.9 (6.1–10.6)	6.3 (5.7–10.6)	
Mean ± SD	8.3 ± 3.1	7.8 ± 3	
Range	2.9–15	2.3–12.6	
T-stadium			0.668 (Pearson Chi-square-test)
Early T-stage (T1+T2)	31 (66%)	29 (62%)	
Advanced T-stage (T3+T4)	16 (34%)	18 (38%)	
N-Stadium			0.301 (Pearson Chi-square-test)
N-negative (N0)	24 (51%)	19 (40%)	
N-positive (N1, N2, N3)	23 (49%)	28 (60%)	
M-Stadium			0.022 (Pearson Chi-square-test)
No metastasis (M0)	47 (100%)	42 (90%)	
Metastasis (M1)	0 (0%)	5 (10%)	
Reconstructive surgery			0.007 (Pearson Chi-square-test)
No flap	22 (65%)	9 (31%)	
Flap	12 (35%)	20 (69%)	

Table 2 shows the T-and N-stages separately with corresponding percentages between the two groups, verifying the roughly equivalent distribution between the COVID-19 pandemic year and before.

**Table 2:** T- and N-stages across the two groups.

	Group A: pre-COVID-19	Group B: COVID-19
T1	15 (32%)	9 (19%)
T2	16 (34%)	20 (43%)
T3	4 (9%)	13 (28%)
T4	12 (25%)	5 (10%)
N0	24 (51%)	19 (40%)
N1	7 (15%)	13 (28%)
N2	9 (19%)	11 (23%)
N3	7 (15%)	4 (9%)

## Discussion

General aspects

Overall, patients’ accessibility to health services, along with their health-seeking behaviour, has been compromised massively during the COVID-19 pandemic for several reasons [3].

The worldwide exponential surge of SARS-CoV-2 infections within a few months at the beginning of 2020 overwhelmed the majority of health systems and altered the clinical routine. Medical and nursing personnel shifted to strengthen the first line of care against the novel virus. Elective surgical procedures were postponed and surgical activity was limited to acute or subacute indications to release anesthesiology capacities for critical care units [10]. Furthermore, medical material shortages [11] and contamination of staff with SARS-CoV-2 [10] constrained the situation even more.

The surgical and interventional workforce adhered to guidelines conscientiously [8] to reduce their exposure to high-risk procedures and protect themselves. Among specialists, otolaryngologists have been at a significantly higher risk of infection due to their proximity to the aerodigestive tract during several procedures [12]. Reports of staff contamination through airborne transmission during sinonasal or tracheal surgery have increased awareness of intraoperative iatrogenic viruses spread through aerosolization [12]. A shift to radiation oncology has been observed in the hopes of preserving precious resources and personnel capacities [13]. Some otolaryngologists have been forced to practice aberrations, such as referring advanced laryngeal cancer patients for primary radiation or favorize more frequent radiation of early laryngeal or human-papilloma-virus-positive (HPV+) pharyngeal carcinoma [13]. Furthermore, some were willing to delay surgery in high-risk patients (age, comorbidities) at high peaks of the pandemic to plan a better therapeutic concept in a safer environment [13].

Governments across the world imposed radical restrictions on social life and mobility freedom, leading to a high burden, anxiety, and uncertainty for patients, who adopted a more self-effacing approach to their health problems; many postponed their regular checkups, neglected follow-ups, and preferred self-treatment strategies for potentially serious symptoms in the face of Covid-19 contamination risk [3]. Inadequate crisis communication confused public opinion and put an extra burden on optimal healthcare.

Oncologic patients have been the focus of attention since the beginning of the pandemic due to the crucial time frame of their therapeutical management [2]. Several guidelines have been published by national associations and international expert panels to avoid fatal delays in oncologic care [6-8]. Studies showed remarkable tumor progression and upstaging of disease in patients with SCC in only four weeks [14]. Therefore, physicians had to find alternative ways of securely providing proper cancer treatment in due time without sacrificing outcomes [2,15]. Nonetheless, oncologic patients are immunocompromised because of surgery or/and systemic radio(chemo)therapy and, thus, are more vulnerable to viral contamination and severe respiratory disease [16]. Lei et al. [17] observed a 20% risk of mortality in patients who unintentionally underwent surgery during the viral incubation period and developed COVID-19. It is obvious that a therapeutic decision has to be made among many controversies and different factors regarding time, resources, and safety for the patient and staff [13].

Current epidemiological data

The impact of the COVID-19 pandemic at the community level was also perceptible. According to a report from the UK, cancer referral cases by general practitioners dropped dramatically [4]. In our cohort, we noticed a slight prolongation (median 15 weeks, not statistically significant) from the first symptom related to the oncologic disease till diagnosis, reflecting limitations in primary care or a change in patients’ behaviour [3]. Significant delays in cancer diagnosis have been observed in Turkey [18] and India [19], indicating compromised accessibility to health services (Table 3). On the other hand, in the USA [20], the median duration of the first visit remained constant at 12 weeks. The interval between DTI remained consistent at about three weeks (median) in our trial and was under the delay threshold reported by a meta-analysis by Graboyes et al. [21]. The same applied to TEI, with a median of approximately 6.5 weeks in both groups. Kiong et al. [20] reported unaffected DTI in a tertiary center in the USA in contrast to cohorts from Heidelberg/Germany, Turkey, and China, where delays occurred [18,22-23] (Table 3).

**Table 3:** Summary of current epidemiological studies concerning impact of COVID-19 pandemic on size and time of diagnosis of head and neck cancer. A: pre-COVID-19 pandemic era, B: COVID-19 pandemic era, pat.: patients, SCC: squamous cell carcinoma.

Article (country)	Comparison periods	Cancer type and localization	Cohort characteristics	Core findings
Laccourreye et al. [24] (France)	1 month	All histologic types	A: 118 pat.	10% decline of inpatient cancer cases
	A: 17.2–17.3.20 vs. B: 17.3–17.4.20	All subsites of head and neck region (incl. paranasal sinuses, salivary gland, thyroid gland, lymphatic tissue, skin)	B: 106 pat.	T3/4 and N2/3 tumors were more frequent in group B
Tevetoğlu et al. [18] (Turkey)	6 months	SCC	A: 60 pat.	T3/4 and N-positive tumors more frequent in group B
	A: 15.3–15.9.19 vs. B: 15.3–15.9.20	Oral cavity, larynx	B: 56 pat.	Time from first symptom to admission increased.
Metzger et al. [22] (Germany)	1 year	SCC	A: 566 pat.	Treatment delay longer in group B
	A: 2010–2019 vs. B: 2020	Oral cavity	B: 58 pat.	Higher T-stage of tumors in group B
Yang et al. [23] (China)	4-weeks	SCC	A: 112 pat.	Time for planning and executing definitive radiotherapy significantly longer
	A: 27.12.19–23.1.20 vs. B: 31.1.-26.2.20	Nasopharynx	B: 82 pat.	
Riju et al. [19] (India)	3 months	Undefined histological type	A: 192 pat.	63 % decline in outpatient and 54% in inpatient visits
	A: 01.4.19–01.3.20 vs. B: 01.4.-30.6.20	Oral cavity	B: 26 pat.	Advanced T-stage more frequent
				More inoperable cases due to advanced stage
Kiong et al [20] (USA)	6 weeks	All histologic types	A: 156 pat.	25% decline in newly diagnosed malignancies
	A: 16.5–20.6.19 vs. B: 14.5–18.6.20	All subsites of head and neck region (incl. paranasal sinuses, salivary gland, thyroid gland, lymphatic tissue, skin)	B: 117 pat.	No delays in symptom-diagnosis and diagnosis-treatment intervals
				Bigger median size of primary tumor in group B
				T3/T4 mucosal tumors more frequent in group B
D'Ascanio et al. [25] (Italy)	1 year	SCC	A: 65 pat.	No significant differences in patients’ cancer stage
	A: 2019 vs. B: 2020	Undefined subsites - included skin cancer and thyroid cancer	B: 56 pat.	

We performed a proportionally similar number of surgical procedures or surgeries followed by adjuvant therapy in both cohorts (Table 1). Whilst elective surgery capacity at our institution radically decreased, we highly prioritized patients with malignancies to deliver fast-track treatment. Furthermore, medical and assistant personnel kept an extraordinary awareness of tumour patients during this period. Unexpectedly, we practiced reconstructive surgery with free tissue transfer and microsurgical anastomosis (for example, radial forearm flap) more often during the pandemic.

This seems paradoxical at first, but there is a possible explanation. At first glance, complex surgery seems to be more resource-demanding and envelope the risk of postoperative critical care [10]. The alternative option of a five-to-six-week chemoradiation regime could, indeed, be riskier for staff and patient contamination due to frequent outpatient visits [13]. Adherence could also be affected in the case of infection and subsequent quarantine, resulting in treatment prolongation and, eventually, a negative outcome. Tevetoglu et al. [18] also performed more reconstructive procedures with pedicled flaps, probably because of the larger initial size (T-stage) of the oropharyngeal tumours. On the contrary, other groups reported a shift to radiation oncology regimes [7,13].

Five out of seven published studies reported more advanced T-and N-stages of HNC patients during the pandemic [18-20,22,24]. Despite severe pressure on health services in northern Italy during the beginning of the virus spread, D'Ascanio et al. [25] did not notice any shift to more advanced cancer stages during this period. These reports, even though geographically limited, indicate the negative side effects of the pandemic on public health. Our data illustrate an unchanged presentation of cancer patients in Berlin; patients’ primary tumor size (T) and locoregional metastases (N) did not occur at higher stages compared to the year before, despite the prolongation from the first symptom to diagnosis.

With the limitation of a small sample size, we observed a statistically significant increase in primary metastatic cases. A closer analysis of these cases revealed that three out of five patients initially presented with a T-stage of T4 (the rest T3 and T2), all of them were N-positive, and had a mean symptom to diagnosis interval of approximately ten weeks. Whilst time to diagnosis is associated with tumor progression and upstaging of disease via locoregional and distant metastases [15,21], we cannot give a viable explanation why metastatic disease occurred more often in our pandemic cohort. A probable causative relationship could be explored in larger trials by considering additional intrinsic factors, like cancer biology and differentiation grade.

Future considerations

The ongoing COVID-19 pandemic has taught us many useful lessons for the future. It highlighted the necessity of establishing pandemic protocols and implementing them rapidly in patient care to minimize risk while maximizing safety and outcome [26]. Adequate personnel and self-protecting equipment are inevitable for the delivery of high-standard health services [12].

It is obvious that we cannot draw final assumptions regarding the impact of the pandemic on the HNC population yet. We definitely need more multi-center, prospective studies to clarify the consequences of the pandemic on a global scale. International networks, such as CovidSurg, offer great platforms for collaboration and contribution in order to produce high-yield clinical data in the COVID-19 era [27]. From now on, we ought to follow up closely with patients who have been diagnosed or treated amidst the pandemic to optimize their outcomes. Future studies should elucidate the recurrence and survival rates of these patients.

Modern technologies and artificial intelligence can contribute to overcoming similar emergency situations. The establishment of telehealth services, such as call consultations or video sessions, is an optimal tool to decongest the overrun outpatient clinics [28]. Automatic risk calculators can rapidly identify suspect cases and refer them for further investigation in the hospital setting [29].

Limitations and bias

In this study, we tried to minimize bias through the inclusion of patients newly diagnosed with HNC, exclusively SCC, in the most common head and neck localizations. Head and neck cancer patients with recurrence or previous malignancy of another kind have been excluded because of their higher awareness and reflexes. The investigation period was comprised of a one-year pandemic and encompassed all possible variabilities: peaks and minimums, within and after the lockdown. The control period was the previous year to enhance comparability. The institutions’ direction, personnel, and organisation remained constant and provided the same level of health services throughout the two-year period. The personnel have been spared from infection by COVID-19.

Nevertheless, the design was mandatory and retrospective. Despite careful examination of the patients’ files, we cannot exclude minor aberrations in subjective clinical data, such as the symptom-diagnosis interval. The monocentric character poses a geographical limitation, and thereafter, conclusions are not applicable nationwide. A subsite-or grading-related data analysis could not be conducted because of the modest number of cases.

## Conclusions

During the COVID-19 pandemic, our data showed consistent diagnostic and therapeutic performance in a tertiary university setting in Berlin, Germany. By highly prioritizing HNC patients' treatment to the detriment of elective cases, we did not notice any significant delays, more advanced primary tumor size, or locoregional metastases in our patients during this period. Nonetheless, these findings reflect a small geographical area, and the current epidemiological data is too sparse to draw conclusions on the real impact of the pandemic on healthcare.
